# Ticagrelor-Induced Dermatological Hypersensitivity Reaction

**DOI:** 10.7759/cureus.85484

**Published:** 2025-06-06

**Authors:** Snehal Lunge, Shimoni R Doshi, Vidyadhar R Sardesai

**Affiliations:** 1 Dermatology, Bharati Vidyapeeth Medical College, Pune, Pune, IND; 2 Dermatology, Venereology and Leprosy, Bharati Vidyapeeth Medical College, Pune, Pune, IND

**Keywords:** : acute coronary syndrome, antiplatelet agents, clopidogrel, drug-induced rash, ticagrelor hypersensitivity

## Abstract

Ticagrelor, a potent antiplatelet medication widely used in acute coronary syndromes, is known for its favourable efficacy and safety profile as compared to clopidogrel. However, skin-related adverse events associated with ticagrelor are rare and not extensively reported in the literature. Here we present a 64-year-old male with a history of coronary artery disease and recent ticagrelor initiation following percutaneous coronary intervention. The patient developed a diffuse erythematous rash involving the trunk and extremities, two days post-ticagrelor initiation, prompting a thorough evaluation for drug-induced hypersensitivity reactions. The rash resolved upon discontinuation of ticagrelor and initiation of clopidogrel therapy along with corticosteroids and antihistamines. This case highlights the importance of recognizing dermatology adverse events associated with ticagrelor.

## Introduction

Ticagrelor, a newer antiplatelet agent introduced in 2011, has gained widespread use following several guideline recommendations favoring it over clopidogrel for the management of acute coronary syndromes [1]. It is the first orally administered P2Y12 receptor antagonist that exerts its effect through reversible binding, in contrast to thienopyridines like clopidogrel that bind irreversibly. This reversible mechanism results in a faster offset of platelet inhibition-typically within three to five days-offering a clinical advantage in scenarios requiring urgent surgical intervention or the management of bleeding complications [2,3]. Common adverse effects of ticagrelor include dyspnoea, bleeding, and bradyarrhythmias. According to the PLATelet inhibition and patient Outcomes (PLATO) trial, dyspnoea occurred in 13.8% of patients on ticagrelor versus 7.8% on clopidogrel; the Prevention of Cardiovascular Events in Patients with Prior Heart Attack Using Ticagrelor Compared to Placebo on a Background of Aspirin (PEGASUS) trial reported dyspnoea in 14.2% on ticagrelor compared to 5.5% on aspirin [1]. However, cutaneous adverse reactions are infrequent, with only a few case reports documenting hypersensitivity skin manifestations following ticagrelor initiation. We present a case report of a 64-year-old male who developed a diffuse erythematous rash over his trunk and extremities secondary to ticagrelor use.

## Case presentation

A 64-year-old Indian man with a past medical history of coronary artery disease, hypertension, ischemic heart disease, and hypothyroidism presented with chief complaints of widespread red, itchy lesions over the trunk for the past two days. The patient reported no prior history of drug allergies or hypersensitivity reactions.

Five days before the rash appeared, he was admitted for Non-ST Segment Elevation Myocardial Infarction (NSTEMI) and underwent transluminal angioplasty of the left anterior descending artery, right coronary artery, and left circumflex artery. He was then started on ticagrelor. After two days of ticagrelor therapy, the patient developed a pruritic maculopapular rash initially localized to his chest, which then generalized over the trunk, predominantly involving the chest and back. The extremities were notably spared, a pattern consistent with delayed-type drug hypersensitivity reactions.

The patient denied any recent use of other new medications, over-the-counter supplements, or exposure to new detergents or soaps. He also denied symptoms including fever, arthralgia, angioedema, blistering, dyspnea, facial edema, difficulty swallowing, or dysuria. These negative findings helped rule out severe hypersensitivity syndromes such as Drug Rash with Eosinophilia and Systemic Symptoms (DRESS), Stevens-Johnson Syndrome (SJS)/Toxic Epidermal Necrolysis (TEN), viral exanthems, and anaphylaxis.

**Figure 1:**
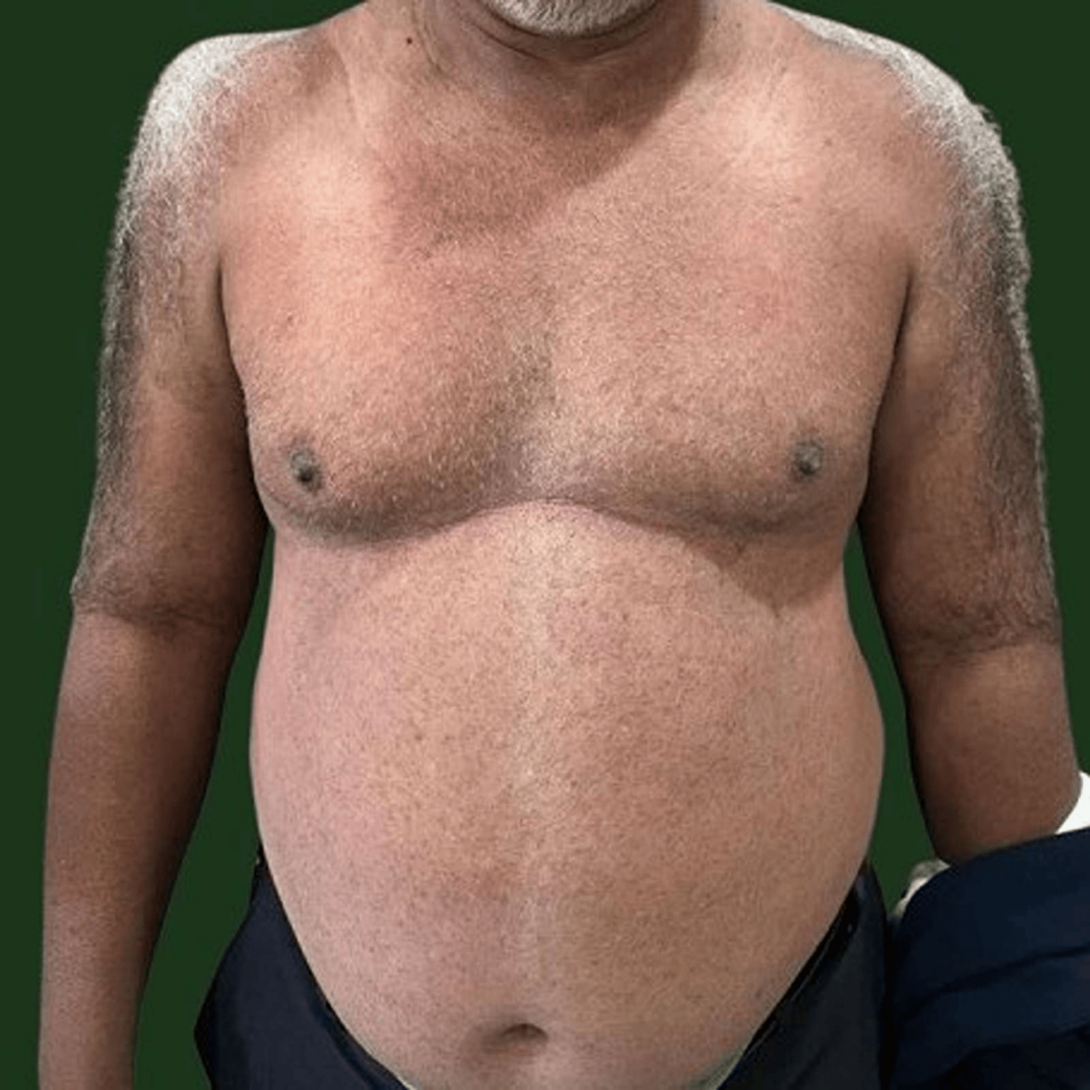
Clinical photograph showing erythematous, blanchable pinpoint papules with a positive diascopy distributed over chest, abdomen.

On examination, erythematous, blanchable, pinpoint papules with positive diascopy were distributed over the chest and abdomen (Figure 1).

**Figure 2:**
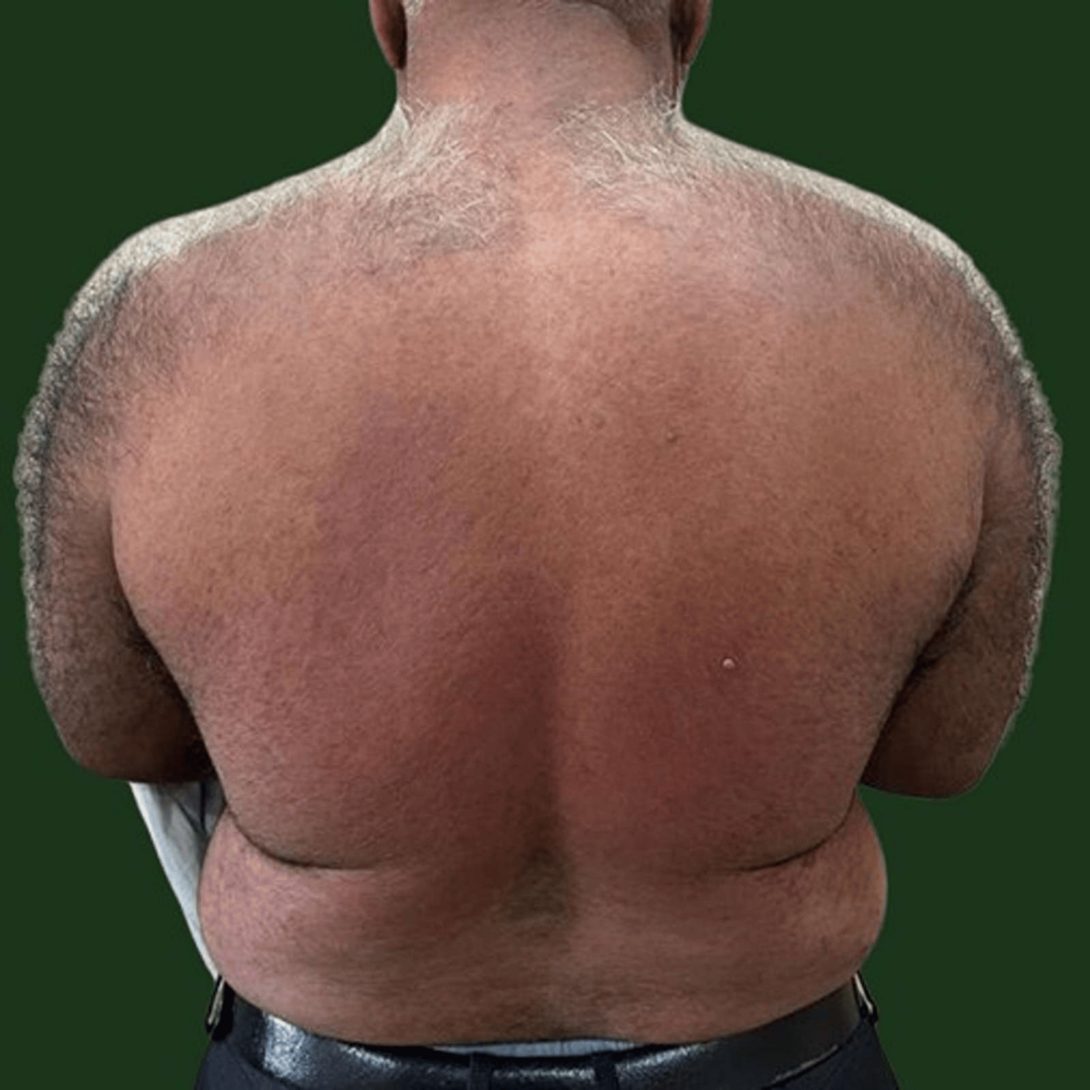
Clinical photograph showing erythematous, blanchable macules, papules coalescing to form plaques over back (predominantly over lumbar region). Diascopy - positive.

Erythematous, blanchable macules and papules coalesced to form plaques over the back, predominantly in the lumbar region, without mucosal involvement (Figure 2).

Baseline investigations and histopathological examination were advised to confirm the diagnosis and exclude other complications; however, the patient declined these investigations.

For management, ticagrelor was discontinued and replaced with clopidogrel, a structurally different P2Y12 inhibitor to maintain antiplatelet therapy while minimizing the risk of cross-reactivity. The patient was started on oral corticosteroids (methylprednisolone 4 mg in tapering doses) to reduce inflammation and an antihistamine (levocetirizine 10 mg) to control pruritus.

## Discussion

Clopidogrel and ticagrelor are antiplatelet medications inhibiting the P2Y12 adenosine diphosphate (ADP) receptor but differ significantly in structure and binding characteristics. P2Y12 ADP receptor blockers are classified into two categories based on their chemical structure: thienopyridines and cyclopentyl-triazole-pyrimidines (CTPs) [1,4]. Ticagrelor, a CTP, binds reversibly to the P2Y12 receptor, whereas clopidogrel, a thienopyridine, binds irreversibly. These structural differences reduce the potential for immunologic cross-reactivity between the two classes, supporting the use of clopidogrel as a suitable alternative in patients with ticagrelor-induced hypersensitivity reactions.

Despite the low structural cross-reactivity, hypersensitivity to ticagrelor may still occur due to idiosyncratic, T-cell-mediated immune responses triggered by the drug or its metabolites. These responses are independent of structural similarity and are influenced by individual immune system characteristics. Ticagrelor-induced skin reactions remain uncommon, with Namazi et al. reporting a 0.6% incidence, and Akdogan et al. identifying only six published cases to date, underscoring the rarity but clinical relevance of these events [5].

Our patient's presentation was similar to previously reported cases by Akdogan et al. and Kawall et al., with a maculopapular rash developing shortly after ticagrelor initiation and resolving upon discontinuation. Notably, unlike reports involving mucosal involvement or bullous lesions, our patient had cutaneous findings only, suggesting a milder clinical course.

Although human leukocyte antigen (HLA) associations are well-described for certain drug reactions, no consistent HLA allele has yet been linked to ticagrelor hypersensitivity [3,4]. The proposed mechanisms of drug reactions involve haptens, formed from the drug or its metabolites, that are presented to naïve T cells, triggering their activation and proliferation. The activated T cells then infiltrate the skin, releasing the inflammatory mediators that contribute to the development of drug-induced rash.

Diagnosis is primarily clinical, and the timing of rash onset can help differentiate the type of drug hypersensitivity reaction. Table 1 summarizes typical timeframes and clinical features of common drug hypersensitivity reactions [6].

**Table 1:** Table summarizing common drug hypersensitivity reactions, their typical onset after drug exposure, and characteristic clinical features to aid in diagnosis and differentiation.

Drug Hypersensitivity Reaction	Typical Onset After Drug Exposure	Clinical Features
Stevens-Johnson Syndrome (SJS) / Toxic Epidermal Necrolysis (TEN)	1–3 weeks	Severe mucocutaneous blistering, epidermal detachment
Drug Rash with Eosinophilia and Systemic Symptoms (DRESS)	2–6 weeks	Fever, rash, eosinophilia, systemic organ involvement
Maculopapular Drug Exanthem	4–21 days	Generalized erythematous maculopapular rash
Acute Generalized Exanthematous Pustulosis (AGEP)	Hours to days	Pustular eruption, fever, neutrophilia
Immediate Hypersensitivity (Type I)	Minutes to hours	Urticaria, angioedema, anaphylaxis

In cases with diagnostic uncertainty or systemic symptoms, further workup may include: complete blood counts (CBC) with differential, liver and renal function tests, antinuclear antibody (ANA) screening to rule out autoimmune mimics, viral serologies, and skin biopsy for histopathological confirmation. If drug hypersensitivity syndrome (DHS) is suspected, criteria such as RegiSCAR can guide diagnosis.

Management includes immediate discontinuation of the suspected drug, switching to an alternative agent, and symptomatic treatment. Antihistamines may relieve pruritus but are not effective in preventing delayed-type, T-cell-mediated hypersensitivity reactions. Desensitization was not considered in our case, as the rash resolved promptly after stopping ticagrelor and switching to clopidogrel. Desensitization is generally reserved for situations where alternative antiplatelet agents are contraindicated or ineffective [3,7].

In our patient, pruritus and a generalized exanthematous rash developed within two days of initiating ticagrelor and improved following withdrawal. This clinical pattern suggests a delayed-type hypersensitivity reaction rather than a severe drug hypersensitivity syndrome (DHS), especially in the absence of systemic symptoms. The rapid onset, resolution on drug cessation, and lack of prior sensitization or systemic involvement support a diagnosis of a simple maculopapular drug eruption.

Clinicians should remain vigilant for early cutaneous signs following ticagrelor initiation. Prompt identification of hypersensitivity allows a timely transition to a structurally unrelated thienopyridine such as clopidogrel, minimizing the risk of cross-reactivity while maintaining necessary antiplatelet therapy to prevent ischemic complications.

## Conclusions

We describe a case of suspected ticagrelor-induced hypersensitivity reaction that was successfully managed conservatively with oral steroids and antihistamines. Given the increasing use of ticagrelor in acute coronary syndrome management, physicians need to recognize these uncommon dermatological manifestations promptly. Although a clear temporal relationship was observed between drug administration and symptom onset with resolution upon discontinuation, the possibility of other etiologies, such as concomitant drug interactions or allergic conditions, cannot be entirely excluded. The absence of histopathological confirmation and investigation highlights the limitations of the single case report. Alternative strategies, such as re-challenge or desensitization, may be explored in scenarios where ticagrelor remains essential. Nevertheless, vigilant pharmacovigilance and timely reporting of such adverse drug reactions remain critical in improving awareness, refining causality assessments, and guiding management. Early identification and intervention can significantly reduce the morbidity associated with hypersensitivity reactions.
